# Polymers for binding of the gram-positive oral pathogen *Streptococcus mutans*

**DOI:** 10.1371/journal.pone.0180087

**Published:** 2017-07-03

**Authors:** Eugene P. Magennis, Nora Francini, Francesca Mastrotto, Rosa Catania, Martin Redhead, Francisco Fernandez-Trillo, David Bradshaw, David Churchley, Klaus Winzer, Cameron Alexander, Giuseppe Mantovani

**Affiliations:** 1 School of Pharmacy, University of Nottingham, Nottingham, United Kingdom; 2 School of Medicine, University of Nottingham, Nottingham, United Kingdom; 3 Department of Pharmaceutical and Pharmacological Science, University of Padova, Padova, Italy; 4 School of Chemistry, Haworth Building, University of Birmingham, Edgbaston, Birmingham, United Kingdom; 5 GlaxoSmithKline, St Georges Avenue, Weybridge, Surrey, United Kingdom; 6 BBSRC/EPSRC Synthetic Biology Research Centre (SBRC), School of Life Sciences, University of Nottingham, Nottingham, United Kingdom; LAAS-CNRS, FRANCE

## Abstract

*Streptococcus mutans* is the most significant pathogenic bacterium implicated in the formation of dental caries and, both directly and indirectly, has been associated with severe conditions such as multiple sclerosis, cerebrovascular and peripheral artery disease. Polymers able to selectively bind *S*. *mutans* and/or inhibit its adhesion to oral tissue in a non-lethal manner would offer possibilities for addressing pathogenicity without selecting for populations resistant against bactericidal agents. In the present work two libraries of 2-(dimethylamino)ethyl methacrylate (pDMAEMA)-based polymers were synthesized with various proportions of either *N*,*N*,*N*-trimethylethanaminium cationic- or sulfobetaine zwitterionic groups. These copolymers where initially tested as potential macromolecular ligands for *S*. *mutans* NCTC 10449, whilst *Escherichia coli* MG1655 was used as Gram-negative control bacteria. pDMAEMA-derived materials with high proportions of zwitterionic repeating units were found to be selective for *S*. *mutans*, in both isolated and *S*. *mutans*–*E*. *coli* mixed bacterial cultures. Fully sulfobetainized pDMAEMA was subsequently found to bind/cluster preferentially Gram-positive *S*. *mutans* and *S*. *aureus* compared to Gram negative *E*. *coli* and *V*. *harveyi*. A key initial stage of *S*. *mutans* pathogenesis involves a lectin-mediated adhesion to the tooth surface, thus the range of potential macromolecular ligands was further expanded by investigating two glycopolymers bearing α-mannopyranoside and β-galactopyranoside pendant units. Results with these polymers indicated that preferential binding to either *S*. *mutans* or *E*. *coli* can be obtained by modulating the glycosylation pattern of the chosen multivalent ligands without incurring unacceptable cytotoxicity in a model gastrointestinal cell line. Overall, our results allowed to identify a structure–property relationship for the potential antimicrobial polymers investigated, and suggest that preferential binding to Gram-positive *S*. *mutans* could be achieved by fine-tuning of the recognition elements in the polymer ligands.

## Introduction

The increasing development of resistance in bacteria to antibiotics is a universal threat to humans and animals,[1] and the detection and inactivation of bacteria thus remains a significant healthcare and societal challenge.[1–3] While much of the focus in addressing infections has been for acute systemic conditions, there are also important healthcare considerations for chronic bacterial diseases, for example those occurring in the oral cavity. The microorganisms found in the human mouth—collectively known as the oral microbiome—include more than 700 bacterial species or phylotypes, a number of which have been associated with conditions such as caries and periodontitis, and several systemic diseases, including bacterial endocarditis, osteomyelitis in children, and cardiovascular disease.[4] Despite a decline in prevalence over recent decades,[5] dental caries in western countries continues to be a significant concern,[1] for example, in the USA dental caries in children are more prevalent than asthma.[6] Furthermore, effective management of dental caries has been shown to improve healthy weight gain, growth rates and overall quality of life of children.[7]

The formation process is similar for all caries. Endogenous bacteria within the biofilm of the tooth produce acids as a result of carbohydrate metabolism, leading to a local drop in pH and demineralisation of the tooth. Current oral hygiene products mostly aim to kill bacteria as their primary mechanism of action.[8, 9] Cariogenic bacteria include *Streptococcus mutans*, *Streptococcus sobrinus* and *Lactobacillus spp*. Among these, *Streptococcus mutans* is the most important, with significance figures of up to P > 0.0001 being quoted between caries and associated *S*. *mutans*.[10] Accordingly, routes to target *S*. *mutans* are of obvious need, with an increasing interest in methods by which the bacteria can be inactivated without invoking selection pressure.

Polymer technology for targeting pathogenic bacteria is being widely explored, and a number of scientific reports and several reviews,[11–14] have been published on this subject. It has been shown that polycations can efficiently kill a variety of bacteria.[12, 15–20] Klibanov and co-workers generated cationic surfaces based on alkylated poly(4-vinylpyridine) (PVP) both by copolymerization of glass-supported acrylamide with 4-vinylpyridine followed by alkylation with a range of 1-alkylbromides (from C3 to C16), and by grafting PVP to an alkylbromide-grafted glass surface, followed by quantitative alkylation of pyridine repeating units with alkyl bromides.[21] These surfaces were assessed for their ability to kill bacteria, which had been sprayed on the surfaces as an aerosol in a manner aimed to replicate actions similar to sneezing or coughing. Under these conditions both these surfaces showed > 90% killing of both Gram-positive *Staphylococcus aureus* and > 99% of *Staphylococcus epidermidis*, and the Gram-negative bacteria *Pseudomonas aeruginosa* and *Escherichia coli*. Whilst these cationic materials are often effective, in the context of oral healthcare indiscriminate sterilisation of the mouth is not always desirable, as commensal bacteria are needed for health preservation by controlling growth of pathogenic microbes.[22, 23] Thus, better prophylactic and therapeutic approaches require selective targeting of harmful organisms while leaving more beneficial species intact. Furthermore, non-lethal means for targeting specific bacteria would offer the additional advantage of preventing the selection of populations resistant against bactericidal agents.[24]

Dental plaque formation and subsequent caries development require an initial step where cariogenic bacteria adhere to the tooth surface. A prerequisite for this process is the adherence of bacteria to the acquired pellicle and subsequent development of oral biofilm, which often occurs through interaction between bacterial lectins and host glycosylated (macro)molecules.[25] Host defence mechanisms include the production of soluble glycoconjugates in saliva, which can offer a first line of defence by competitively blocking bacterial adhesion proteins. Thus, biomimetic approaches which include analogous anti-adhesion glycosylated molecules could be potentially utilized to modulate the virulence of oral pathogens.

The ability of *S*. *mutans* to bind to glycans with galactosyl residues has been described in several studies.[25–28] Reduction of salivary galactosylated *O*–glycans has been associated with increased caries susceptibility in children,[29] whilst early work by Nagata et. al. showed that daily mouth rinsing with 9% D-galactose solution could inhibit the formation of dental plaque,[30] thus suggesting that appropriately designed galactose-containing ligands would have potential to prevent adhesion of *S*. *mutans* to oral surfaces. Lectin binding affinities can be significantly increased when multivalent sugar ligands are utilized,[31, 32] a phenomenon known as *cluster glycoside* effect.[33] Thus, multivalent carbohydrate ligands could potentially be utilized for binding /sequestration of *S*. *mutans* if suitable ligand architecture and sugar motifs were identified.

Bacteria differ in their surface chemical and structural features, and species-selective binding has been demonstrated using a number of methods, for example utilizing charge interactions,[3, 34] saccharide-based binding agents,[35–38] and cell structural mimics through templating[39–41] and antibiotics.[42] We recently showed that multivalent polymeric ligands can be engineered through a ‘bacteria-instructed synthesis’ where ligands are generated by using bacterial cell surfaces as templates, hijacking bacterial metal-binding and redox pathways to activate the required polymerization catalysts.[3] Polymer ligands are often used for bacterial binding studies due to their potential to span over receptors and chemical functionalities at the cell surface, and bind them in a multivalent fashion.[31, 32, 43, 44]

In this work we designed two libraries of synthetic polyvalent ligands—with either cationic/zwitterion or carbohydrate binding units—and tested them for their ability to bind selectively the pathogenic *S*. *mutans* oral bacteria. In this proof-of-principle study, readily available model Gram-negative *E*. *coli* bacterium, which has also been found in the oral microflora of periodontitis patients[45–47] and *Vibrio harveyi* were used for comparative experiments, while *Staphylococcus aureus*, often found in the oral cavity and perioral region, and associated with a range of pathological conditions,[48] was chosen as a model Gram-positive bacterium. Chemistries on the polymer ligands were varied to target both charged functionalities and adhesion lectins at the bacterial surface. This initial study indicates that both approaches can lead to preferential binding to specific pathogenic bacteria, and suggest that the chemistry of macromolecular synthetic ligands must be modulated on a case by case basis to optimise the interactions between dynamic natural surfaces and synthetic materials.

## Materials

Chemicals: 2-(Dimethylamino)ethyl methacrylate (DMAEMA, 97)%, 1, 3-propane sultone (99%), 2-bromoethanol (97%), and ethylenediamine (99%) were purchased from Alfa Aesar. Copper (I) Bromide (99.999%), α-bromoisobutyryl bromide (98%), 4-(dimethylamino)pyridine (DMAP, 99%) and *N*-ethyl-*N’*-(dimethylaminopropyl)carbodiimide hydrochloride (EDC, commercial grade), LB (Lysogeny Broth) medium, hydrogen chloride solution 2 M in diethyl ether, alizarin red S (AR-S), 3-(trimethylsilyl)propargyl alcohol (97%), D-glucose, 2,4 dihydroxybenzaldehyde (98%) and *N*-acetyl glycine (99%) were obtained from Sigma Aldrich. Benzyl alcohol (99%), iodomethane (99%), coumarin 343 (laser grade) and oxalyl chloride (98%) were purchased from Acros Organics. All the chemicals were used as received without further purification, unless otherwise stated. Dulbecco’s phosphate-buffered saline (PBS) without Ca^2+^ and Mg^2+^ was purchased from Lonza. Caco2 Media was Dulbecco’s Modified Eagle Medium supplemented with 2 mM L-glutamine, 1000 IU/mL penicillin, 10 μg/mL streptomycin and 10% foetal bovine serum (FBS).

Bacteria: *Escherichia coli* (*E*. *coli*) MG1655, *Staphylococcus aureus* (*S*. *aureus)* SH1000, and *Vibrio harveyi* (*V*. *harveyi*) BB170 were obtained from stocks held within the University of Nottingham and *Streptococcus mutans* (*S*. *mutans*) NCTC 10449 was provided by GlaxoSmithKline Consumer Healthcare. *Streptococcus mutans* was cultured in brain heart infusion (BHI) while *E*. *coli* and S. aureus were grown in LB broth at 37°C, and *V*. *harveyi* in LB broth at 30°C. Primary cultures were grown overnight without agitation and the main bacterial cultures were grown by mixing at 200 rpm.

### Instrumentation

Mass Spectra (MS) (TOF-ESI) were recorded on a Waters 2795 separation module/Micromass LCT KC453 platform, under positive or negative scan mode where applicable. Purified compounds were directly injected using OpenLynx software.

Nuclear magnetic resonance (NMR) spectroscopy was carried out on the Bruker DPX400 Ultra-Shield^™^. Chemical shifts are reported in ppm (units) downfield from internal tetramethylsilane (TMS) or from the signal of deuterated solvents used. Analysis of spectra was done using MestReNova 6.0.2 copyright 2009 Mestrelab Research S. L.

Aqueous Size Exclusion Chromatography (SEC) was performed on a Polymer Labs GPC50 Plus fitted with differential refractometer (RI), capillary viscometer (DP) and dual angle laser light-scattering (15 and 90) detectors. The eluent was Dulbecco's PBS without Ca^2+^ and Mg^2+^, at 30°C and a flow rate of 1.0 mL min^-1^. The instrument was fitted with a Polymer Labs aquagel-OH guard column (50 × 7.5 mm, 8 μm) followed by a pair of PL aquagel-OH columns (30 and 40, 300 × 7.5 mm, 8 μm). Calibration for detector response and inter-detector delays was achieved using a single, narrow PEO standard (Polymer Labs, *M*_p_ 128 kDa, [η] 1.2968 dL g^-1^) using a dn/dc value of 0.133 g mL^-1^.

Analysis of cationic polymers was carried out on a Shimadzu Prominence UPLC system fitted with a differential refractive index detector. The mobile phase was 1 M acetic acid with 0.3 M NaH_2_PO_4_ (pH 3.0) at a flow rate of 1.0 mL min^-1^. The columns used were the same as used for the aqueous SEC system and calibrated using single, narrow poly(2-vinylpyridine) standards (Polymer Standard Services, Germany) of known molecular weight (0.8–256 kDa). Poly(2-(dimethylamino)ethyl) methacrylate was analyzed on the same GPC50 Plus system, equipped with Resipore Mixed-D columns. The mobile phase was a 5% trimethylamine solution in choroform at a flow rate of 1.0 mL min^-1^. Molecular weight was calculated based on a standard calibration method using polystyrene standards EasiVial PS-M, Agilent Tech. Glycopolymers were analyzed on GPC plus system equipped with Agilent PLgel 5 μm Mixed D columns. The mobile phase was DMF + 0.1% w/w LiBr at flow rate of 1.0 mL min^-1^. Molecular weights and polydispersity indices were calculated based on a standard calibration method using PMMA narrow standards (505–1,810,000 g mol^-1^).

Infrared analysis was carried out using Thermo Scientific Nicolet IR 200 FT-IR. Analysis of spectra was done using Omnic 8.0 (copyright 1992–2008 Thermo Fisher Scientific Inc).

Bacterial analysis and images were produced using a NIKON EFD3 microscope and a NIKON DXM1200 digital camera. Software used was ACT-1 2.7 copyright NIKON. Image analysis was carried out using ImageJ 1.49k. Bacterial cluster size was determined by laser diffraction using a Coulter LS230 particle size analyser (Beckman Coulter, High Wycombe, UK).

### Methods

Synthesis of benzyl-2-bromo-2-methylpropanoate: the ATRP initiator was produced according to a method previously described in the literature.[43, 49] Benzyl alcohol (15.0 mL, 145 mmol) and triethylamine (16.3 mL, 117 mmol) were dissolved in dichloromethane (150 mL) in a round-bottomed flask, and the resulting solution was cooled to 0°C using an ice bath. To this, α-bromoisobutyryl bromide (16.3 mL, 132 mmol) was added dropwise over a period of 30 minutes and then left to stir at this temperature for two hours. The mixture was washed with water (8 x 50 mL) and then with a saturated sodium bicarbonate aqueous solution (2 x 50 mL). The organic layer was dried over magnesium sulfate, filtered, and the volatiles were removed under reduced pressure to yield a yellow oily residue which was purified by vacuum distillation to yield analytically pure benzyl-2-bromo-2-methylpropanoate as a clear colourless liquid (20.98 g, 81.59 mmol, 62% yield).

^1^H NMR (400 MHz, CDCl_3_) δ (ppm): 2.01 (s, 6H, C*H*_*3*_), 5.26 (s, 2H, C*H*_*2*_O), 7.51–7.30 (m, 5H, C*H*_aryl_). ^13^C {^1^H}-NMR (100 MHz, CDCl_3_) δ (ppm): 30.85 (2C, *C*H_3_), 55.86 (1C, *C*CH_3_), 67.55 (1C, *C*H_2_), 127.96, 128.40, 128.67 (5C, *C*_aryl_), 135.56 (1C, *C*_aryl_), 171.32 (1C, *C*O). FTIR (neat): ν 2970 (broad), 1730, 1460, 1390, 1270, 1160, 1100, 735, 696 cm^-1^. Expected m/z for (M-H)^+^: 257.0172. Found 257.1235.

Polymerization of 2-(dimethylamino)ethyl methacrylate (DMAEMA): 2-(Dimethylamino)ethyl methacrylate (DMAEMA; 20.0 g, 127 mmol), (E)-*N*–(pyridine-2-ylmethylene)propan-1-amine (397 μL, 2.54 mmol) and benzyl-2-bromo-2-methylpropanoate (327 mg, 1.27 mmol) were added into a Schlenk tube along with toluene (20 mL). The vessel was sealed with a rubber septum and subjected to five freeze-pump-thaw cycles to degas the mixture. At the end the mixture was left frozen and flushed with nitrogen before Cu(I)Br (182 mg, 1.27 mmol) was added. The system was then subjected to three nitrogen/vacuum cycles, the reactor was filled back with nitrogen and the temperature adjusted to 70°C with constant stirring (t = 0). Aliquots were removed at regular time intervals for monitoring of conversion, which was calculated by ^1^H NMR by comparing the relative proportion of C(O)OC*H*_2_ protons at 4.19 and 4.36 ppm for the polymer and residual monomer, respectively. The polymerization was stopped at 93% conversion by cooling the reaction mixture to room temperature and exposing it to air. The mixture was then passed through two neutral alumina pads to remove the residual Cu(II) salts, eluting with additional toluene. The solution volume was reduced under reduced pressure, and the pDMAEMA polymer **(1)** was isolated as a colourless viscous oil by precipitation into petroleum ether. The signals of the benzyl group at the polymer chain-end were found to be too small to allow a reliable estimation of M_n_ by ^1^H NMR, hence in this work M_n,SEC_ was utilized instead.

DP_SEC_ = 107. M_n,SEC_ = 17.1 kDa, Đ_SEC_ = 1.18; conversion 93%.

#### Polymer labelling

Synthesis of coumarin 343 2’-bromoethyl ester: coumarin 343 was modified using a method previously described in the literature for an analogous compound.[49]

A solution of coumarin 343 (0.285 g, 1.00 mmol) and bromoethanol (354 μL, 5.00 mmol) in CH_2_Cl_2_ (10 mL) was cooled to 0°C using an ice bath, and *N*-ethyl-*N'*-3-dimethylaminopropyl)carbodiimide hydrochloride (EDC, 0.575 g, 3.00 mmol) and 4-(dimethylamino)pyridine (DMAP, 0.006 g, 0.05 mmol) were sequentially added under stirring. The resulting mixture was left to react overnight, then washed with water (4x50 mL) and dried over MgSO_4_. After filtration the solvent was removed under reduced pressure and the resulting residue purified by flash chromatography (CC, SiO_2_, dichloromethane/ethyl acetate 12:1 vol/vol). The relevant fractions were combined and the solvent removed under reduced pressure to give coumarin 343 2’-bromoethyl ester as an orange crystalline solid (0.0972 g, 0.248 mmol, 25% yield).

^1^H NMR (400 MHz, CDCl_3_) δ (ppm) = 1.89–2.04 (m, 4H, 2CH_2_), 2.76 (t, J = 6.0 Hz, 2H, CH_2_), 2.87 (t, *J* = 6.4 Hz, 2H, CH_2_), 3.28–3.37 (m, 4H, CH_2_N), 3.63 (t, *J* = 6.3 Hz, 2H, CH_2_Br), 4.58 (t, *J* = 6.3 Hz, 2H, CH_2_O), 6.95 (s, 1H, CH_vinyl_), 8.35 (s, 1H, CH_aryl_). FTIR (neat): ν 2960, 2920, 2852, 2356, 2341, 1725, 1444, 1409, 1257, 1085, 1010, 788, 698 cm^-1^.

Fluorescent labelling of poly(2-(dimethylamino)ethyl methacrylate: a suspension of pDMAEMA **(1)** (3.19 g, 20.3 mmol of tertiary amine repeating units), coumarin 343 2’-bromoethyl ester (0.079 g, 0.20 mmol) and potassium iodide (0.337 g, 2.03 mmol) in acetone (70 mL) was ultrasonicated for 30 minutes to aid dissolution of reagents. This mixture was then allowed to stir at 45°C for 2 days and at 55°C for further 2 days, while monitoring the conversion by thin layer chromatography (TLC) using chloroform as the mobile phase, following the disappearance of the coumarin alkyl bromide, and the increase of fluorescence in the polymer spot at the baseline. When coumarin 343 2’-bromoethyl ester could no longer be detected, the solvent was removed under reduced pressure, and the mixture redissolved in CH_2_Cl_2_. After filtration coumarin 343-tagged polymer **(2)** (2.44 g, 15.2 mmol, yield: 75%) was collected by precipitation into petroleum ether.

Alternatively, pDMAEMA **(2)** (250 mg, 1.59 mmol of tertiary amine repeating units) was dissolved in dichloromethane (10 mL). Most of the solvent was removed under reduced pressure (the residue had the consistence of a viscous oil), and acetone (10 mL) was added. To the resulting solution, coumarin 343 2’-bromoethyl ester (0.0064 g, 0.016 mmol) and potassium iodide (0.0072 g, 0.048 mmol) in acetone (10 mL) were added. The solvent was removed under reduced pressure, and the resulting residue was heated in the dark without additional solvent nor stirring, at 60°C for 24 h. The resulting residue was utilised for the following steps without further purification.

Modification of pDMAEMA: varying degrees of DMAEMA quaternization [50] and sulfobetainization [51] were obtained using methods previously reported in the literature.

Synthesis of *N*,*N*,*N*-trimethylethanaminium quaternized cationic polymers (3)_25-100%_: to quaternize fluorescent pDMAEMA **(2)**, four vessels were prepared containing polymer **(2)** (0.200 g, 1.27 mmol of tertiary amine repeating units) in each, then tetrahydrofuran (8.2 mL) was added. After dissolution, methyl iodide was added under stirring in varying amounts: for 25% target quaternization 19.8 μL (0.318 mmol); for 50% conversion 39.6 μL (0.636 mmol); for 75% conversion 59.4 μL (0.954 mmol) and for 100% conversion 79.2 μL (1.27 mmol) were added. The resulting mixtures were stirred for 48 hours to yield a precipitate of an amber colour. The solvent was removed under reduced pressure, and the residues were dissolved in deionized water and precipitated in THF. The solid residue was collected by centrifugation, dissolved in water, and the resulting yellow solutions were frozen and freeze-dried in the dark for 7 days. The experimental degree of quaternization was estimated by ^1^H NMR, by comparing the integral of the CH_2_N signal of the unmodified repeating units at 2.66 ppm, and that of the analogous residue in the quaternized repeating units at 3.75 ppm. Results are summarized in Table 1.

**Table 1:** Experimental degrees of quaternization and sulfobetainization of fluorescent pDMAEMA (2).

	Polymer (code)	Targeted modification (%)	Calculated modification^a^ (%)
**Quaternized (3)**	**(3)**_**25%**_	25	26
	**(3)**_**50%**_	50	55
	**(3)**_**75%**_	75	80
	**(3)**_**100%**_	100	100
**Sulfobetainized (4)**	**(4)**_**25%**_	25	30
	**(4)**_**50%**_	50	56
	**(4)**_**75%**_	75	68
	**(4)**_**100%**_	100	100

^a^Degree of modification, defined as the percentage of polymer repeating units modified following quaternization with methyl iodide or 1,3-propane sultone (sulfobetainization), was quantified by ^1^H NMR. Calculated modification percentages include 1% modification with coumarin 343 2’-bromoethyl ester.

Synthesis of sulfobetaine zwitterionic polymers (4)_25-100%_: for the sulfobetaination reactions, four vessels were prepared containing the fluorescent pDMAEMA **(2)** (0.200 g, 1.27 mmol) which was dissolved in tetrahydrofuran (8.2 mL for each flask). While stirring, 1,3-propane sultone was added in varying amounts. For 25% conversion 27.9 μL (0.318 mmol), for 50% conversion 55.8 μL (0.636 mmol), for 75% conversion 83.7 μL (0.954 mmol), and for 100% conversion 112 μL (1.27 mmol) were added. The resulting reaction mixtures were stirred for 16 hours at room temperature, during which the formation of a waxy yellow precipitate was observed. The solvent was removed under vacuum and the residues dissolved in deionized water and precipitated in THF. The solid residue was collected by centrifugation, dissolved in water, and the resulting yellow solutions were frozen and freeze-dried for 7 days. The experimental degree of sulfobetaination was estimated by ^1^H NMR by comparing the integral of the CH_2_N signal of the unmodified repeating units at 2.66 ppm, and that of the analogous residue in the sulfobetainated repeating units at 3.75 ppm. Results are summarised in Table 1.

Synthesis of fluorescent mannosylated **(5)**_**Man**_ and galactosylated **(5)**_**Gal**_ glycopolymers: The mannosylated **(5)**_**Man**_ and galactosylated **(5)**_**Gal**_ polymers used were synthesized following a protocol adapted from Ladmiral *et al*. [49] as described in the S1 File.

Mannosylated glycopolymer **(5)**_**Man**_ DP = 73 M_n,SEC_ = 26 kDa, Đ_SEC_ = 1.31.

Galactosylated glycopolymer **(5)**_**Gal**_ DP = 73 M_n,SEC_ = 22 kDa, Đ_SEC_ = 1.28.

Colocalization analysis: colocalization of polymer and bacteria was analyzed with ImageJ 1.49k. Before processing, images were recorded for each light wavelength separately and afterwards examined with the JACoP plug-in.[52] For each full image, background fluorescence was subtracted using a rolling ball radius of 50 pixels. Pearson’s coefficients were calculated using the plug-in and used to assess the co-localization.

Polymer binding experiments: the ability of the polymers produced in this study to bind *E*. *coli*, *S*. *mutans*, *S*. *aureus*, and *V*. *harveyi* was assessed following a protocol described by Rawlinson *et al*. [50] Briefly, a 1.00 mL aliquot of 1.0 mg mL^-1^ polymer solution was prepared in sterile deionized water. This solution was incubated with bacteria with OD_600_ = 0.1 of for 30 minutes. After this the bacteria were pelleted and the pellet was washed three times with phosphate buffer solution (PBS) to remove unbound polymer. After washing, the pellet was resuspended in PBS and mounted on microscope slides to be viewed. The same procedure was carried out with a water blank solution to serve as the control. Photographic images were generated using phase contrast and fluorescence microscopy (10,000x).

Size distributions of bacteria-polymer clusters: the analysis was carried out as described previously by Magennis *et*. *al*.[3] 400 μL of a bacterial suspension with an OD_600_ of 1.3 were added to the flow cell to obtain an obscuration of 8–12%, as per the manufacturer’s recommendation. Samples were prepared by mixing 400 μL of bacterial suspension and 100 μL of a 10 mg/mL solution of **(4)**_**100%**_, the suspension was allowed to equilibrate for 30 minutes under constant stirring. At this point the population distribution was recorded with constant mixing. Control samples were prepared using bacteria only, with no added polymer.

Statistical analysis: statistical analysis was carried out by calculating a two way ANOVA in Prism^®^ Version 6.05. Images were imported into ImageJ 1.49k then converted into an 8-bit format. This image was threshold-adjusted and the intensities of fluorescence found as a percentage of the region of analysis. Means and standard deviations were calculated in Excel for each sample type and the mean of the total sample set was used to calculate the two-way ANOVA.

Cell viability tests. 3-(4,5-dimethylthiazol-2-yl)-2,5-diphenyltetrazolium bromide (MTT) assay: into a 96 well plate, 0.100 mL of Caco-2 cells suspended in Dulbecco’s Modified Eagle Medium was pipetted, such that each well contained 15,000 cells. Cells were incubated at 37°C for 20–24 hours to allow cell-surface attachment. Serial dilutions of each polymer solution were performed with supplemented Dulbecco’s Modified Eagle Medium to obtain a final concentration range of 0.0050–10 mg mL^-1^ for quaternized and sulfobetainized DMAEMA based polymers and 0.010–5.0 mg mL^-1^ for glycopolymers **(5)**_**Man**_ and **(5)**_**Gal**_. These solutions were sterile filtered and incubated at 37°C in media overnight to check for bacterial growth and confirm sterility. Once the cells had attached, the media was removed from the wells and 0.100 mL of the polymer-media solution was added leaving one lane of untreated cells and a further lane of cells treated with 1% Triton X-100 (TX) in media. The plate was incubated for a further 24 hours at 37°C after which the media was removed and the cells were washed once with PBS. Then, 0.100 mL of 1.0 mg mL^-1^ MTT reagent in media was added to each well, and the plate protected from light by wrapping it in aluminium foil. The plate was incubated for 2 hours at 37°C and then the media was removed and 0.200 mL of isopropanol were added, and the plate was agitated on an orbital shaker for 5 minutes. Using a Tecan plate reader, the absorbance at λ = 570 nm was recorded from each well, and the values averaged across the polymer concentration replicates. The percentage of MTT metabolized was normalized according to equation 1.

$$\text{\% MTTmetabolized }=\;\frac{\text{(treatmentgroup avg}.\;-\text{ TX avg}.)*100}{\text{Untreatedcells avg}.\;-\text{ TX avg}.}$$

## Results and discussion

### Synthesis of bacteria-binding macromolecular ligands

Initial experiments focused on generating polymers able to interact with negatively charged functionalities—e.g. teichoic acids for Gram-positive bacteria, and charged LPS and phospholipids in the outer membranes of Gram-negative bacteria[34, 53] at the surface of the pathogens.

**Fig 1:**
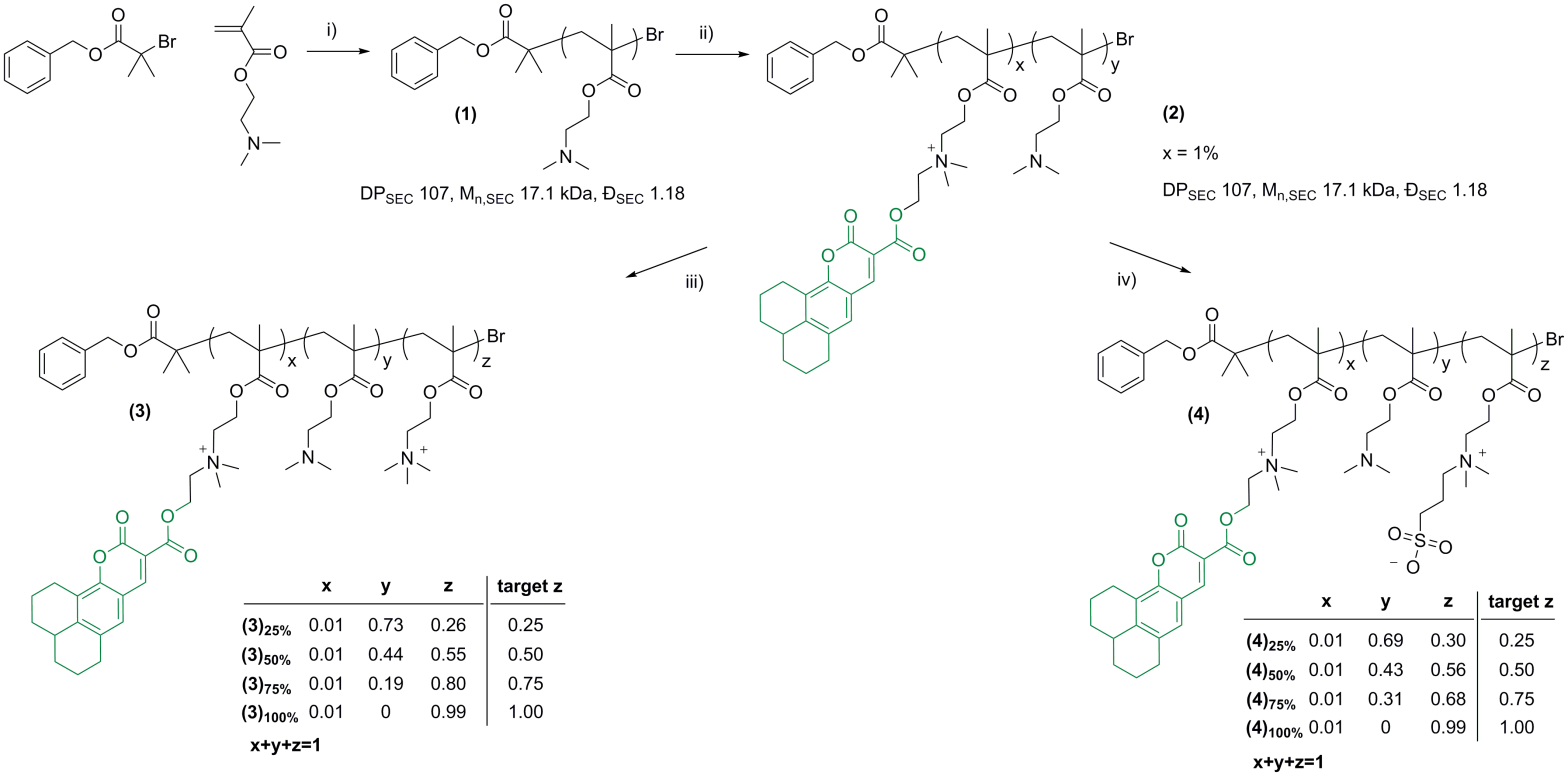
Generation of fluorescent quaternized pDMAEMA libraries (3) and (4). *Reagents and conditions*: i) CuBr, (E)-*N*-(pyridine-2-ylmethylene)propan-1-amine, 70°C toluene; ii) Coumarin 343 2’-bromoethyl ester, potassium iodide, acetone, 45 to 55°C. iii) THF, methyl iodide. Initial (polymer tertiary amino groups): (methyl iodide): 1:0.25, 1:0.5, 1:0.75, 1:1 mol:mol, iv) THF, 1,3-propane sultone. Initial [polymer tertiary amino groups]_0_: [1,3-propane sultone]_0_: 1:0.25, 1:0.5, 1:0.75, 1:1 mol:mol.

To investigate the impact of charged macromolecular ligands on binding to Gram-positive *S*. *mutans* and Gram-negative *E*. *coli*, initially a library of polymers possessing different degrees of charged repeating units was generated by polymerization of 2-(dimethylamino)ethyl methacrylate (DMAEMA) *via* conventional Atom Transfer Radical Polymerization (ATRP) followed by post-polymerization modification of the resulting (DMAEMA)_107_ **(1)** polymer precursor (Fig 1). The DMAEMA tertiary amino group possesses a pKa of 8.4, while commonly lower values are reported for its polymer.[54, 55] Therefore, pDMAEMA polymers are expected to be mostly positively charged at physiological pH.

Post-polymerization modification of the precursor polymer pDMAEMA was carried out by quaternization with either methyl iodide, to obtain cationic *N*,*N*,*N*-trimethylethanaminium iodide groups, or 1,3-propane sultone, to generate zwitterionic sulfobetaine moieties (for clarity this process is termed here simply as sulfobetainization). The resulting library of polymers shared a common backbone—that is, all polymers in this library had the same number of repeating units—whilst they differed for the nature of the functional pendant groups, their proportion with respect of the total number of polymer repeating units, or both (Table 1 and Fig 1). Having all potential macromolecular ligands with the same chain length was important for this comparative study, as the extent by which multivalent ligands interact with functionalities located at cell membrane is often dependent on the ligand macromolecular size.[32] Modification of pDMAEMA has previously been explored to modify its interaction with selected bacteria, for example to generate non-fouling surfaces on poly(dimethylsiloxane).[56] Firstly, pDMAEMA **(1)** was fluorescently tagged with coumarin 343 with 2’-bromoethyl ester (1% with respect of DMAEMA repeating units) in acetone in the presence of NaI, to facilitate the detection of polymer ligands in subsequent bacterial binding assays. The resulting coumarin 343-tagged pDMAEMA **(2)** (λ_ex.max_ 441 nm, λ_em,max_ 480 nm) was utilized as a common precursor for cationic and zwitterionic ligands **(3)**_**25-100%**_ and **(4)**_**25-100%**_, respectively. The degree of polymer functionalisation was tuned by simply varying the molar ratio between DMAEMA tertiary amine repeating units, and either methyl iodide or 1,3-propane sultone, in the reaction feed, resulting in percentages of targeted quaternization or sulfobetainization ranging from 25 to 100% (Table 1 and Fig 1). The final degree of modification was determined by ^1^H NMR analysis of the obtained polymers **(3)**_**25-100%**_ and **(4)**_**25-100%**_, by comparing the integral of signal for the two protons adjacent to the tertiary amine (NCH_2_-, 2.66 ppm) with that of the same two protons adjacent to the modified amine moieties (^+^NCH_2_-, 3.75 ppm). The experimental degrees of modification were found to be in good agreement with the expected values, indicating the protocol optimised for these reactions allowed near-quantitative conversion of methyl iodide and 1,3-propane sultone into the corresponding quaternized and sulfobetainizedsulfobetainized polymer repeating units, respectively (Fig 1).

**Fig 2:**
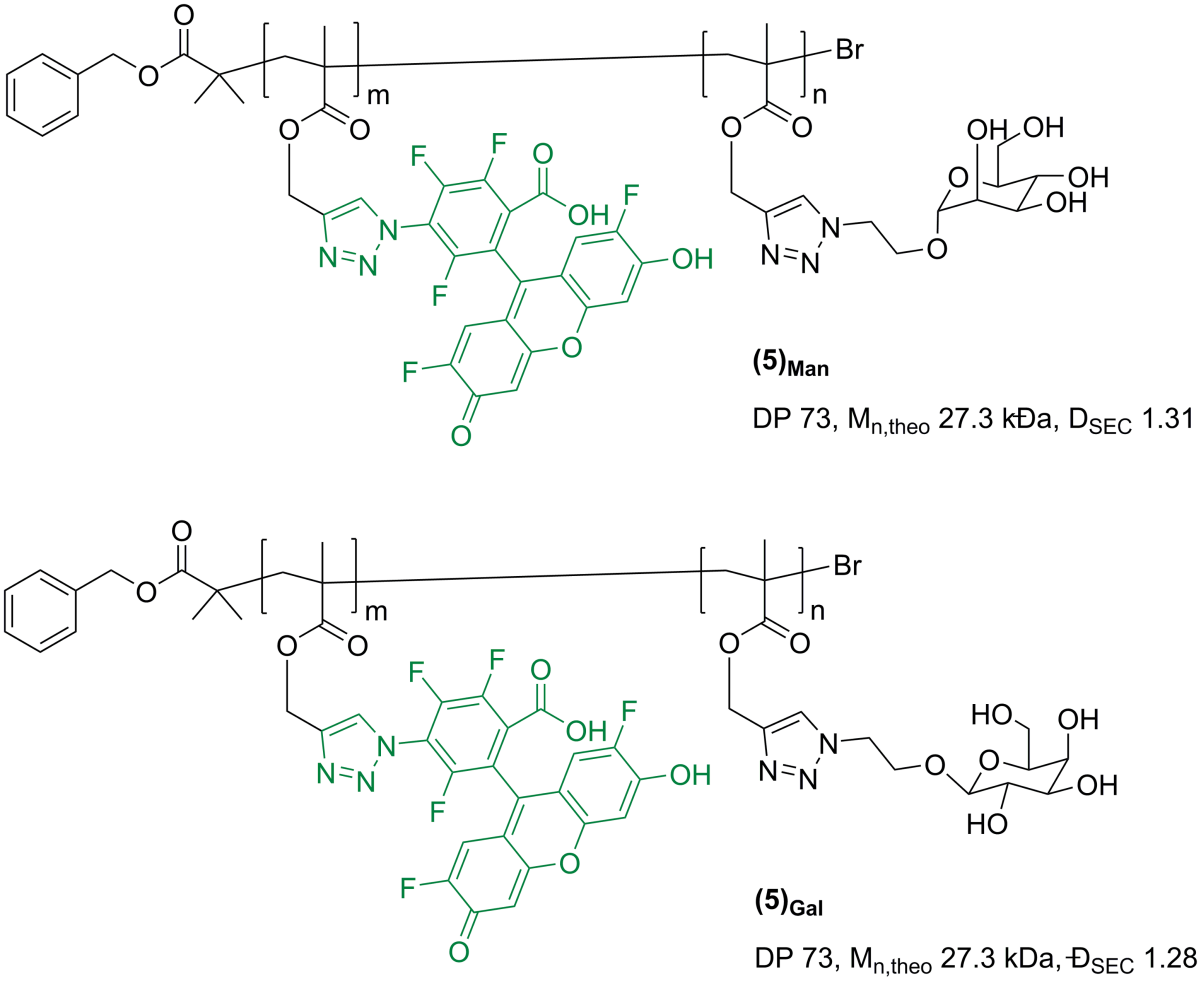
Mannosylated (5)_Man_ and galactosylated (5)_Gal_ glycopolymers used in this study.

To explore the possibility of selectively sequester *S*. *mutans* by targeting its carbohydrate-recognizing machinery, a smaller sub-family of glycosylated ligands was also prepared. Synthetic glycopolymers possessing specific sugar pendant units in recent years have received increasing attention due to their ability to mimic a range of natural lectin-binding glycans.[57–61] Accordingly, galactosylated glycopolymer **(5)**_**Gal**_ (Fig 2) was synthesized and investigated as a potential multivalent ligand for *S*. *mutans* Gal-binding adhesins.

*S*. *mutans* is known to be able to bind also to non Gal-based sugar ligands—e.g. a range of glucans, via GbpA-D adhesins[62]–thus mannosylated glycopolymer **(5)**_**Man**_ was also prepared and tested. Polymers were synthsised following a procedure developed by us and Haddleton[49] which involves first the assembly of a poly(propargyl methacrylate) reactive polymer intermediate by ATRP, which was then functionalised with the required sugar motifs by copper-catalysed azide-alkyne cycloladdition (CuAAC), using either 2’-azidoethyl-*O*-galactopyranoside or 2’-azidoethyl azidoethyl-*O*-mannopyranoside in DMF, at ambient temperature, for 72 h (see S1 File). As for the synthesis of cationic and zwitterionic ligands **(3)**_**25-100%**_ and **(4)**_**25-100%**_, glycopolymers **(5)**_**Man**_ and **(5)**_**Gal**_ were prepared by functionalisation of a preformed polymer precursor, which allowed to obtain two multivalent ligands featuring identical macromolecular size and architecture, and only differed for the nature of the sugar binding units. An Oregon Green fluorescent tag was inserted in the glycopolymer structure to facilitate detection in subsequent bacteria binding studies.

### *Bacteria-*polymer binding studies: Cationic vs. zwitterionic macromolecular ligands

The binding of the polymer ligands **(3)**_**25-100%**_ and **(4)**_**25-100%**_ to target pathogenic bacteria *S*. *mutans* NCTC 10449 was investigated using both fluorescence and phase contrast microscopy. As a control, polymer binding to *E*. *coli* MG1655 under identical conditions was also tested, and the results were compared with those obtained for the cariogenic *S*. *mutans*. *E*. *coli* was selected as, although commonly associated with urogenital and gastrointestinal infections, it has also been detected in high prevalence and levels in periodontal sites of chronic periodontitis patients.[45–47]

In these initial tests, polymers **(3)**_**25-100%**_ and **(4)**_**25-100%**_ (1.0 mg mL^-1^ in deionized water) were incubated with bacteria at OD_600_ = 0.1 of bacteria for 30 min and after removal of the excess of fluorescent macroligands, polymer-bacteria interaction was assessed by phase contrast and fluorescence microscopy. Binding was expected to result in bacterial aggregation, through cross-linking between charged polymer-covered regions on bacteria cell walls and other complementarily charged bacterial cells. Binding affinities were estimated from the ability of each polymer ligand to induce bacterial aggregation as has been previously reported to estimate affinity of macromolecular ligands to bacterial cells.[3, 34, 37] However, it should be noted that the correlation between binding affinity and aggregate formation is not always quantitative, as it includes a kinetic component which can vary with cell concentration. To overcome this potential limitation, the measurement of polymer fluorescence established a second means for quantification of binding.

**Fig 3:**
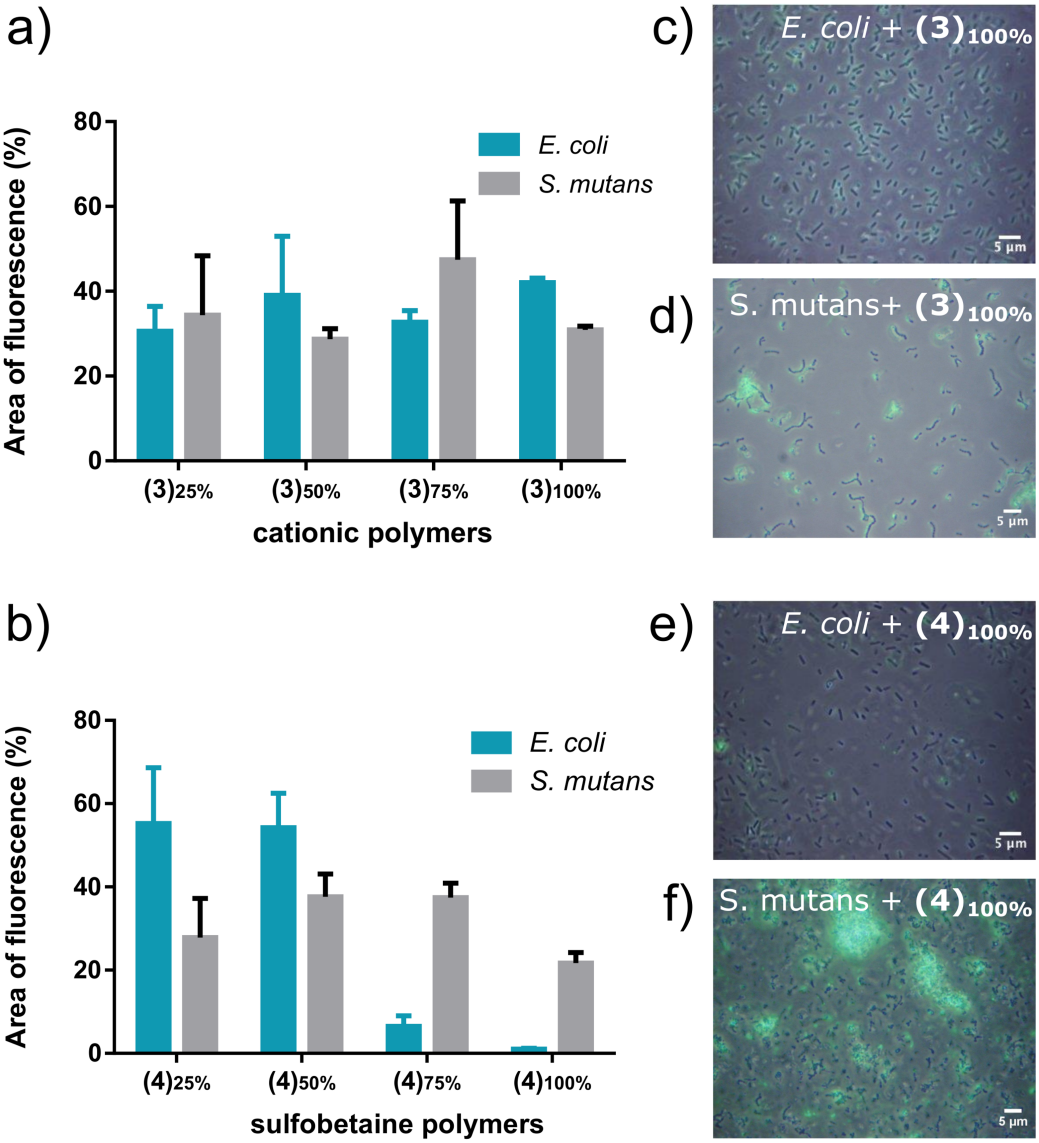
Binding of coumarin 343-tagged a) cationic **(3)_25-100%_** and b) sulfobetaine **(4)_25-100%_** polymers at different degrees of functionalization, to *E*. *coli* and *S*. *mutans* in bacterial suspensions of OD_600_ 0.1, and 1.0 mg mL^-1^ polymer solutions. Area of fluorescence (%) was quantified using ImageJ. Error bars represent standard deviations on independent experiments (N = 3). Fluorescence micrographs are shown for fully functionalised (c, d) cationic and (e, f) sulfobetaine polymers, **(3)**_**100%**_ and **(4)**_**100%**_, respectively using the 488 nm (green) channel.

In this study *N*,*N*,*N*-trimethylethanaminium quaternized cationic materials **(3)**_**25-100%**_ showed efficient binding of both *S*. *mutans* and *E*. *coli* species, at all degrees of quaternizations investigated (Fig 3a).

These results were expected, as positively charged polymers are known to bind different bacterial species through electrostatic interactions with negatively charged bacterial cell walls[63, 64] and they have been employed to bind a range of bacteria species which include *Vibrio harveyi*,[65] *Staphylococcus aureus*,[34] *Pseudomonas aeruginosa*,[66] and *E*. *coli*.[67] However, sulfobetaine-containing polymers **(4)**_**25-100%**_ displayed remarkably different binding profiles. Data showed that, whilst binding to *E*. *coli* dramatically decreased as the proportion of zwitterionic sulfobetaine repeating groups increased, with almost no bacterial adhesion detected for **(4)**_**100%**_ (Fig 3b and 3e), that of *S*. *mutans* was high and essentially constant at all degrees of functionalisation, (Fig 3b and 3f).

To further investigate the binding ability of zwitterionic polymethacrylates to a range of different bacterial strains, fully sulfobetainated **(4)**_**100%**_ was selected. Gram-positive *Staphylococcus aureus*, frequently isolated in the oral cavity and perioral region,[68, 69] where it can contribute to a range of conditions which include angular cheilitis, parotitis, and staphylococcal mucositis,[48] and Gram-negative *Vibro harveyi* were included in this study. While the latter is not a human pathogen *per se*, other species of the genus Vibrio such as *Vibrio cholerae* and *Vibrio parahaemolyticus* are important human pathogens of immediate concern regarding infection.[34]

**Fig 4:**
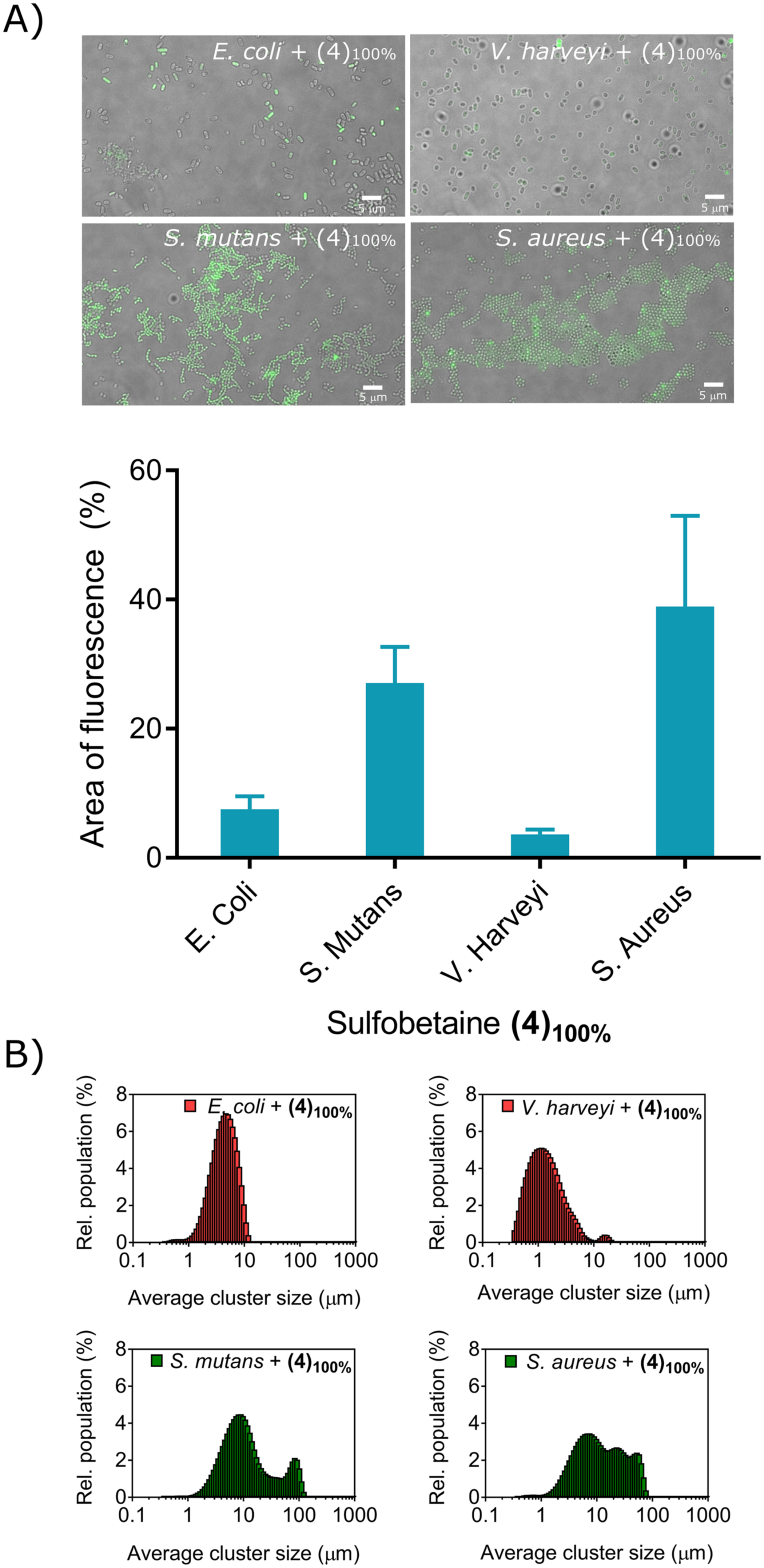
(A) Binding of coumarin 343-tagged sulfobetaine polymer **(4)**_**100%**_ to *E*. *coli*, *S*. *mutans*, *V*. *Harveyi* and *S*. *Aureus* in bacterial suspensions of OD_600_ 0.1, and 1.0 mg mL^-1^ polymer solutions (scale bars = 5 μm). Representative fluorescence micrographs are shown using the green channel (488 nm excitation). Area of fluorescence (%) was quantified using ImageJ. Error bars represent standard deviations of three equivalent areas on three different micrographs. (B) Bacterial aggregation mediated by sulfobetaine polymer **(4)**_**100%**_, as quantified *via* master sizer (Coulter counter) analysis of polymer—bacteria clusters.

Sulfobetaine zwitterionic polymer **(4)**_**100%**_ were found to selectively bind for Gram-positive *S*. *mutans* and *S*. *aureus* (Fig 4A) which was rather intriguing, as zwitterionic polymers are frequently utilized as anti-fouling materials,[70–73] due to their ability to form tightly bound water layers. This association of polyzwitterions with water occurs through strong ionic interactions such that disruption of the water-polymer interfacial layer is entropically unfavourable. In this way the polymers prevent protein deposition and subsequent biofilm development. *S*. *mutans* produces most of the exopolysaccharides that are commonly found in dental biofilms.[74] It has been recently demonstrated that extracellular polysaccharides can enable bacteria to colonize zwitterionic surfaces which are resistant to protein conditioning.[75, 76] The utilization of zwitterionic materials to prevent biofouling is well reported in the literature[70, 71, 77] yet despite this, the nature of the interaction between soluble zwitterionic polymers with polysaccharide-encased bacteria has yet to be explored.

Evident bacterial clustering of *S*. *mutans* and *S*. *aureus* was observed upon treatment with **(4)**_**100%**_, as observed with formation of large visible aggregates, whereas only individual bacterial cells or small clusters (≤ 10 μm) could be observed for *V*. *harveyi* and *E*. *coli*, respectively (Fig 4B and Figure E in S1 File). Within these settings, polymers mediate bacterial clustering by first binding to the exterior surface of bacterial cells, then by promoting adhesion of polymer-coated bacteria to non-polymer-coated surfaces of other bacteria.[3] Importantly, results from these clustering experiments followed the same trends previously observed in the fluorescence experiments, showing that extensive polymer binding promotes subsequent bacterial aggregation, thus further validating the results obtained in the initial fluorescence microscopy experiments.

**Fig 5:**
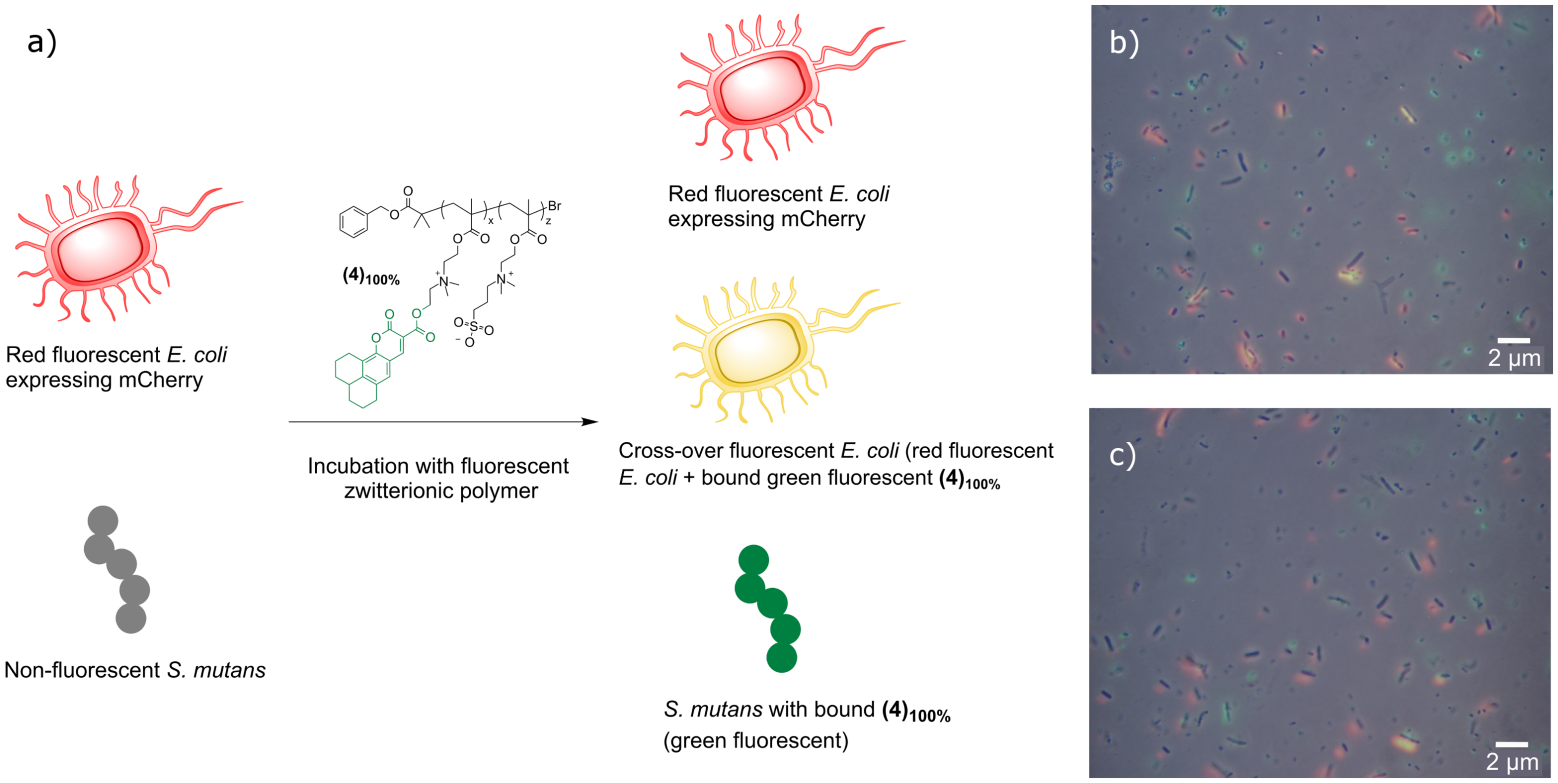
Selective polymer binding in mixed bacterial cultures: schematic representation of bacteria cross-over experiment. a) Bacteria with an OD_600_ = 0.1 were mixed with 1.0 mg mL^-1^ of polymer in deionized water and incubated for 30 minutes before washing with PBS three times, re-suspending in PBS and mounting 10 μL for microscopic imaging. b) and c) Overlapped fluorescence microscopy images of mixed culture experiment. Images recorded sequentially, then overlapped. Experiments were performed in duplicate (b) and c)).

These data showed that zwitterionic macromolecular ligands with high sulfobetaine content might allow selective binding of *S*. *mutans* in specific mixed bacterial cultures (Fig 5a). Accordingly, *S*. *mutans* and red fluorescence emitting *E*. *coli* MG1655 mCherry were mixed at equal cell densities (OD_600_ = 0.1) and incubated with the fully betaine-modified fluorescent polymer **(4)**_**100%**,_ at a concentration of 1.0 mg mL^-1^ in deionized water. Selective binding to *S*. *mutans* was indeed demonstrated, where green coumarin 343 **(4)**_**100%**_-*S*. *mutans* complexes were clearly visible (bacteria were non-fluorescent, whilst the polymer ligand possessed a green fluorescent tag), and minimal fluorescence cross-over was detected for the green polymer and red fluorescent *E*. *coli* (Fig 5b and 5c).

To validate this visual observation a co-localization analysis was carried to determine the overlap of red fluorescence emitted by mCherry and green fluorescence emitted by the polymer coumarin 343 label. The Pearson's Coefficient values calculated (Fig 3b and 3c) were r = 0.190 and 0.122, respectively. The Pearson correlation coefficient is a measure of the degree of co-localization or correlation of fluorescence between fluorophores within a micrograph.[78] A value close to zero indicates no correlation and a value ± 1.0 indicates a positive or negative correlation.[52] The analyses of the mixed culture experiments demonstrate that there were negligible areas of fluorescence cross-over, which could be explained in terms of either non-specific binding of the polymer to *E*. *coli* or spatial proximity between red mCherry *E*. *coli* bacterial cells and green coumarin 343 **(4)**_**100%**_-*S*. *mutans* complexes. Taken together, these results indicated that zwitterionic polymers were able to act as partially selective ligands for Gram-positive for *S*. *mutans* and *S*. *aureus* bacteria.

### *S*. *mutans* and *E*. *coli* polymer binding studies: Gal vs. Man glycopolymer ligands

The ability for *E*. *coli* to bind mannosylated glycans *via* FimH sub-units in Type 1 fimbriae is well documented[79] and mannosylated glycopolymers have been utilized for its detection or sequestration.[37, 80, 81] However, *E*. *coli* is also known for binding other sugar molecules—e.g. Gal-containing residues through P-fimbriae specific for galabiose (α-D-Gal-(1→4)-β-D-Gal) disaccharide in glycosphingolipids.[80] In this study the potential for preferential binding and sequestration of *E*. *coli* was therefore again investigated using mannosylated **(5)**_**Man**_ and galactosylated **(5)**_**Gal**_ glycopolymer ligands.

**Fig 6:**
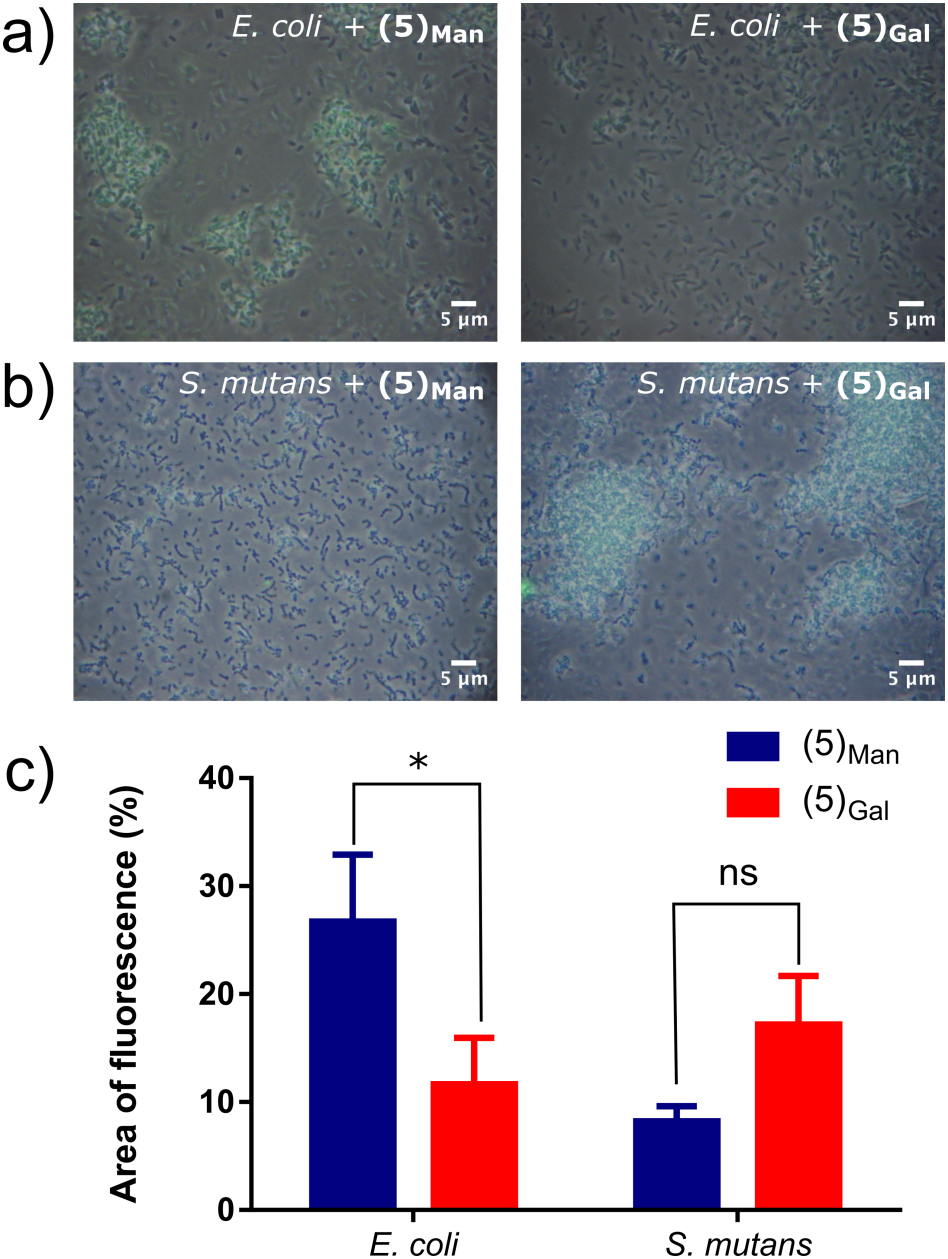
Top) Micrographs of *E*. *coli* and *S*. *mutans* after incubation with mannose **(5)**_**Man**_ and galactose **(5)**_**Gal**_ fluorescent glycopolymers, a) *E*. *coli* and b) *S*. *mutans* after incubation with Oregon Green-tagged glycopolymers **(5)**_**Man**_ (left) and **(5)**_**Gal**_ (right) at a concentration of 1.0 mg mL^-1^. Experiments were run in triplicate. c) Percentage of fluorescence per microscope image, at an OD_600_ of 0.1. Fluorescence was determined using ImageJ by converting into 8-bit then thresholding the images and quantifying percentage area of fluorescence.

Accordingly, solutions of Oregon Green-tagged **(5)**_**Gal**_ and (**5)**_**Man**_ (1.00 mg mL^-1^ in deionized water) were incubated with *E*. *coli* and *S*. *mutans* (10^9^ colony forming units (CFU) of bacteria) for 30 minutes, and after removal of the excess of fluorescent glycopolymers, binding was probed using microscopy (Fig 6).

As expected from previous studies which utilized mannose-containing polymers[44, 82], polymer **(5)**_**Man**_ strongly bound *E*. *coli*, thus resulting in a relevant fluorescence signal detected (Fig 6a, left image) whereas significantly less binding was observed for galactose glycopolymer (**5)**_**Gal**_ (Fig 6a, right image). Binding of Gal-presenting polymers to *E*. *coli* has been described before,[38] although in some cases expression of bacterial lectins can be strain-specific. The opposite trend was observed for *S*. *mutans*, where higher avidity was observed for galactose glycopolymer (**5)**_**Gal**_ (Fig 6b, right image) compared to its mannosylated analogue (Fig 6b, left image). This is in line with previous work by Schüler *et al*., which described specificity of *S*. *mutans* strains for glycans presenting terminal galactose residues.[25]

Similarly to the charged polymers, the significance between differences in binding were tested using a two way ANOVA incorporating Tukey’s multiple comparison statistical test. P values were calculated for both inter-species and inter-saccharide analysis. Significant differences were found for *E*. *coli* binding to mannosylated and galactosylated polymers (P = 0.026), whereas the difference for *S*. *mutans* lay just beyond significance with a P value of 0.064, suggesting that a degree of preferential binding to *S*. *mutans* using the galactosylated ligand **(5)**_**Gal**_ could be achieved.

### Cytocompatibility assays

**Fig 7:**
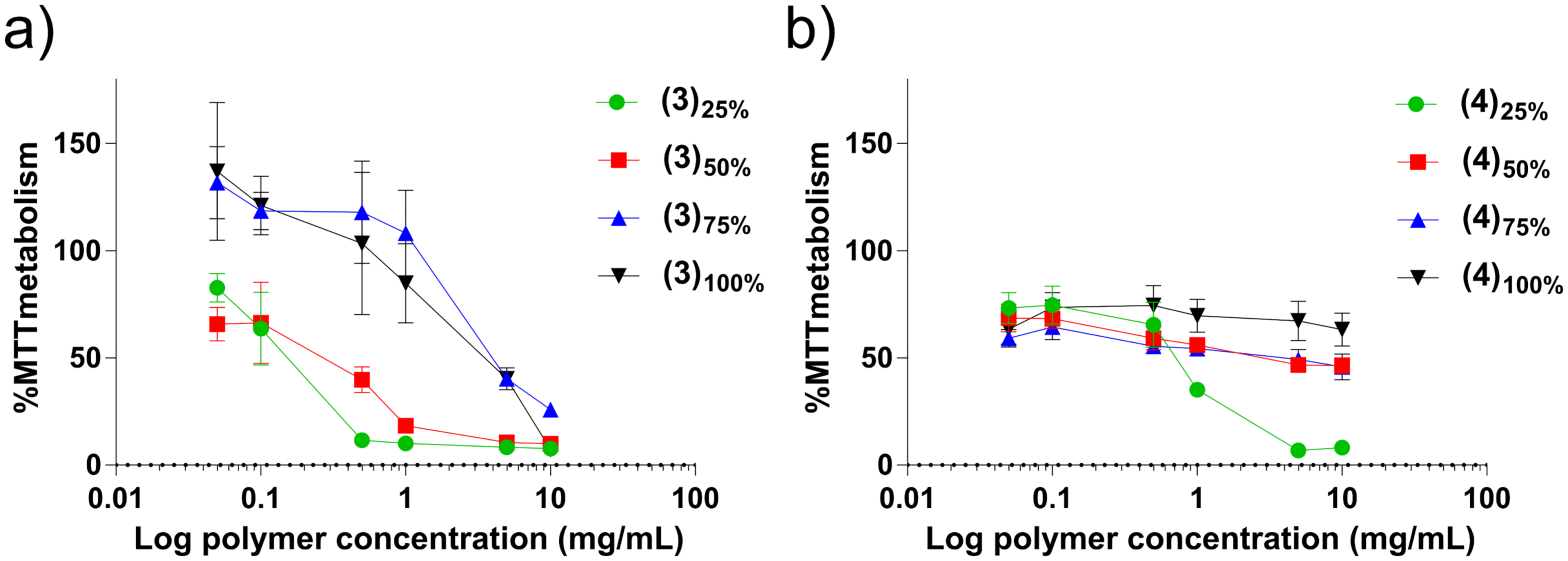
MTT assay results performed on Caco-2 cell line for a) quaternized and b) sulfobetaine based pDMAEMA polymers (green circle ●) 25% modified, (red squares ■) 50% modified, (blue triangles ▲) 75% modified and (black triangles ▼) 100% modified in a concentration range 0.050–10 mg mL^-1^ for 24 h. Cell viability is expressed as a percentage of MTT metabolized compared to control untreated cells. Experiments were performed in triplicate.

To assess how the degree of substitution to pDMAEMA, either as trimethyl-alkyl ammonium salts or sulfobetain zwitterions, affects the cytotoxicity of the resulting materials, polymers **(3)**_**25-100%**_ and **(4)**_**25-100%**_, were incubated with the Caco-2 human epithelial cell line for 24 h, and an MTT assay was then performed. This cell line was chosen as it is well characterised and frequently used to assess toxicity of compounds in the gastrointestinal tract.[83] Toxicity was assessed in a 0.050–10.0 mg mL^-1^ polymer concentration range (Fig 7).

In agreement with previous studies,[84–87] cationic polymers displayed a considerable and concentration-dependent toxicity against human cells. It has also been noted that even at low doses the polymers exert a stress response upon the cells, causing increased MTT metabolism.[88, 89] Indeed, the toxicity associated with positively charged polymers is one of the major limitation which prevents their clinical applications.[85] Conversely, sulfobetaine-derivatised pDMAEMA **(4)**_**25-100%**_ polymers showed a significantly lower toxicity levels against human epithelial cells, especially at high content in zwitterionic repeating units. Zwitterionic polymers are typically characterised by low toxicity[77, 90] and have been employed in hydrogel formulations, demonstrating excellent biocompatibility.[91] As already discussed, free tertiary amines of pDMAEMA are mostly protonated at physiological pH, thus for the zwitterionic pDMAEMA **(4)**_**25-100%**_polymer sub-library, lower degrees of modification result in higher proportions of protonated free tertiary amines, which confer a partially polycationic character to the polymers, which resulted in increased cytotoxicity as evident for polymer **(4)**_**25%**_. Importantly, for the other zwitterionic polymers, no significant cytotoxicity was observed even at very high polymer concentrations (10 mg mL^-1^).

**Fig 8:**
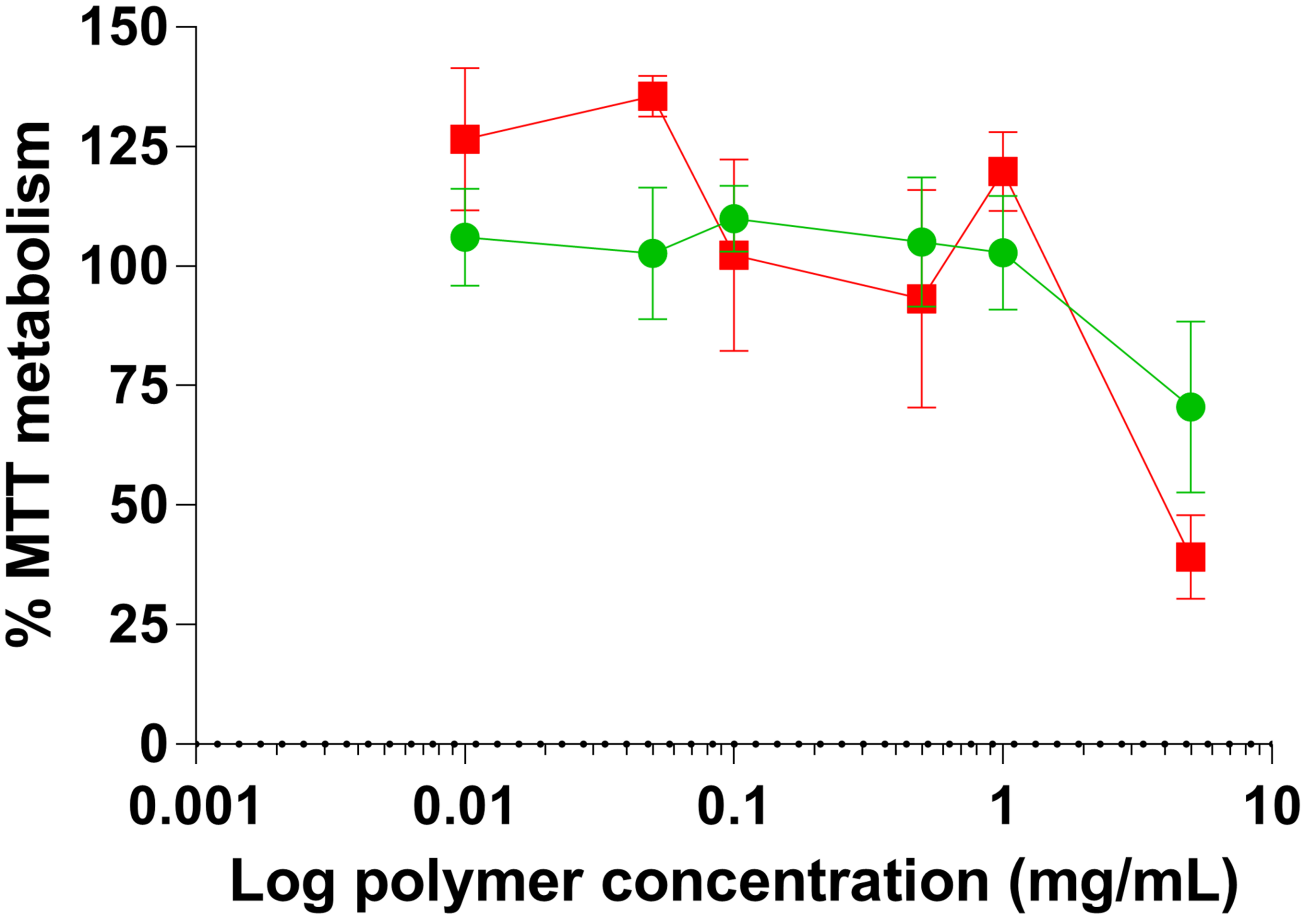
Metabolic activity of Caco-2 cell line incubated with (red squares ■) galactosylated (5)_Gal_ or (green circles ●) mannosylated (5)_Man_ glycopolymers for 24 hours in a concentration range 0.010–5.0 mg mL^-1^ for 24 h. Cell viability is expressed as a percentage of MTT metabolized by a control group of untreated cells. Experiments were performed in triplicate.

Cytocompatibility of the glycopolymers **(5)**_**Man**_ and **(5)**_**Gal**_ were assessed in the same manner as for the charged polymers. The metabolism of MTT was measured as concentration of the polymers was increased over a concentration range of 0.010–5.0 mg mL^-1^ (Fig 8).

At low concentrations cell viability was unaffected and metabolism of MTT was similar to that of the negative control group. At the highest concentration tested (5.0 mg mL^-1^) the toxicity increased for both polymers in an analogous manner irrespective of the nature of the sugar pendant moieties, a phenomenon that has been also described for other glycopolymers.[92, 93] No cytotoxicity was observed up to a relatively high polymer concentration (1.0 mg mL^-1^) for both galactosylated **(5)**_**Gal**_ or mannosylated **(5)**_**Man**_ glycopolymers.

## Conclusions

In this study a range of cationic, zwitterionic, and glycosylated polymers were investigated as potential ligands for the preferential sequestration of Gram-positive oral pathogen *S*. *mutans*. Using Gram-negative *E*. *coli* as control, initial bacterial binding experiments showed that, unlike cationic copolymers containing *N*,*N*,*N*-trimethylethanaminium groups, the ability of sulfobetaine-containing materials to bind *E*. *coli* decreased dramatically as the proportion of zwitterionic repeating units increased. However, binding to *S*. *mutans* target bacteria using these polymer ligands was retained, suggesting that preferential binding of this oral pathogen could be achieved. Subsequent experiments with fully sulfobetainized that included a wider range of bacterial strains, showed preferential binding towards Gram-positive *S*. *mutans* and *S*. *aureus*, compared to Gram-negative *E*. *coli* and *V*. *harveyi*. Importantly, subsequent binding experiments using coumarin 343-labelled zwitterionic polymers **(4)**_**100%**_ in the presence of both *S*. *mutans* and red fluorescence emitting *Escherichia coli* MG1655 mCherry, exhibited preferential/exclusive binding to the former, thus showing the ability of **(4)**_**100%**_ to give selective bacterial recognition in mixed bacterial cultures. More synthetically complex glycopolymer-based ligands were evaluated as an additional class of potential sequestrants, and results showed preferential interaction of mannosylated and galactosylated macromolecules for *E*. *coli* and *S*. *mutans*, respectively.

Taken together these results suggest that polymeric materials could be developed as potential non-antibiotic antimicrobials for cariogenic *S*. *mutans* when chemistries on the polymer ligands are further developed to optimise bacterial binding.

## Supporting information

S1 File Describes the synthesis and characterization of glycopolymers (5)_Gal_ and (5)_Man_, and corresponding intermediates, and additional polymer-mediated bacterial aggregation data. **Figure A**: Synthetic route of mannosylated and galactosylated glycopolymers via ATRP. Reagent and conditions: a) *N*-(ethyl)-2-pyridylmethanimine/Cu(I)Br, toluene, 70°C; b)TBAF, acetic acid, THF, -20°C to RT; c) Cu(I)Br, bipyridine, oregon green azide, ascorbic acid, DMF, 72 hours, RT; d) 2’-azidoethyl-*O*-galactopyranoside or 2’-azidoethyl azidoethyl-*O*-mannopyranoside, DMF, 72 hours, RT. **Figure B**: Synthesis of 6-azido-2,4,5,7,7’-pentafluorofluorescein. *Reagents and conditions*: i) CH_3_SO_3_H, reflux, 48 h; (ii) NaN_3_, water/acetone 1:5, 50°C. **Figure C**: ^1^H NMR purified galactosylated Oregon Green-labelled glycopolymer **(5)**_**Gal**_ in DMSO-d_6_. **Figure D**: ^1^H NMR of purified mannosylated Oregon Green-labelled glycopolymer **(5)**_**Man**_ in DMSO-d_6_. **Figure E**: Polymer-mediated bacterial aggregation as quantified *via* master sizer (Coulter counter) analysis of polymer—bacteria clusters: treatment with sulfobetaine polymer **(4)**_**100%**_ (right column) *vs*. untreated bacteria controls (left column).
(DOCX)
