# Supplementary material for: Polymers for binding of the gram-positive oral pathogen Streptococcus mutans
Source: PLoS One. 2017 Jul 3;12(7):e0180087. doi: 10.1371/journal.pone.0180087 (PMC5495209; doi:10.1371/journal.pone.0180087)
Supplement: S1 File — Figure A: Synthetic route of mannosylated and galactosylated glycopolymers via ATRP. Reagent and conditions: a) N-(ethyl)-2-pyridylmethanimine/Cu(I)Br, toluene, 70°C; b)TBAF, acetic acid, THF, -20°C to RT; c) Cu(I)Br, bipyridine, oregon green azide, ascorbic acid, DMF, 72 hours, RT; d) 2’-azidoethyl-O-galactopyranoside or 2’-azidoethyl azidoethyl-O-mannopyranoside, DMF, 72 hours, RT. Figure B: Synthesis of 6-azido-2,4,5,7,7’-pentafluorofluorescein. Reagents and conditions: i) CH3SO3H, reflux, 48 h; (ii) NaN3, water/acetone 1:5, 50°C. Figure C: 1H NMR purified galactosylated Oregon Green-labelled glycopolymer (5)Gal in DMSO-d6. Figure D: 1H NMR of purified mannosylated Oregon Green-labelled glycopolymer (5)Man in DMSO-d6. Figure E: Polymer-mediated bacterial aggregation as quantified via master sizer (Coulter counter) analysis of polymer—bacteria clusters: treatment with sulfobetaine polymer (4)100% (right column) vs. untreated bacteria controls (left column). (DOCX) [file pone.0180087.s001.docx]

**Electronic Supporting Information**

**Polymers for binding of the Gram-positive oral pathogen *Streptococcus mutans*.**

*Eugene P. Magennis,^1^ Nora Francini,^2^ Francesca Mastrotto,^1,3^ Rosa Catania,^1^ Martin Redhead,^1^ Francisco Fernandez-Trillo,^4^ David Bradshaw,^5^ David Churchley,^5^ Klaus Winzer,^6^ Cameron Alexander,^1,^* and Giuseppe Mantovani.^1,^**

^1^ School of Pharmacy, University of Nottingham, Nottingham NG7 2RD, UK.

^2^ School of Medicine, University of Nottingham, Nottingham NG7 2UH, UK.

^3^ Department of Pharmaceutical and Pharmacological Science, University of Padova, Via F. Marzolo 5, 35131 Padova, Italy.

^4^ School of Chemistry, Haworth Building, University of Birmingham, Edgbaston,

Birmingham B15 2TT, UK.

^5^ GlaxoSmithKline, St Georges Avenue, Weybridge KT13 0DE, Surrey, UK.

^6^ BBSRC/EPSRC Synthetic Biology Research Centre (SBRC), School of Life Sciences, University Park, University of Nottingham, Nottingham, UK.

1. **Synthesis**

Mannosylated and galactosylated glycopolymers were synthesized by a slight modification of a previously described procedure (1). Oregon green azide was utilized as labelling agent to obtain fluorescent glycopolymers.

**Figure A.** **Synthetic route of mannosylated and galactosylated glycopolymers via ATRP.** Reagent and conditions: a) *N*-(ethyl)-2-pyridylmethanimine/Cu(I)Br, toluene, 70°C; b)TBAF, acetic acid, THF, -20°C to RT; c) Cu(I)Br, bipyridine, oregon green azide, ascorbic acid, DMF, 72 hours, RT; d) 2’-azidoethyl-*O*-galactopyranoside or 2’-azidoethyl azidoethyl-*O*-mannopyranoside , DMF, 72 hours, RT.

Clickable poly(propargyl methacrylate) was prepared as described by Ladmiral *et al*,(1) using the following reaction conditions: [(I)]:[Monomer]:[Cu(I)Br]:[Ligand] = 1:65:1:2; benzyl-2-bromo-2-methylpropanoate initiator (I): 40.2 mg; trimethylsilylpropargylmethacrylate (Monomer) 2.00 g, Cu(I)Br: 22.5 mg, *N*-(ethyl)-2-pyridylmethanimine (Ligand): 42.1 mg;, toluene 5 mL, 70°C. Conversion 80%; initiating efficiency = 71.2 %.

**Synthesis of 6-azido-2,4,5,7,7’-pentafluorofluorescein (Oregon Green azide).**

Oregon green azide was synthesised via a two-step synthetic pathway.

**Figure B.** **Synthesis of 6-azido-2,4,5,7,7’-pentafluorofluorescein.** *Reagents and conditions:* i) CH_3_SO_3_H, reflux, 48 h; (ii) NaN_3_, water/acetone 1:5, 50°C.

2,4,5,7,7’-hexafluorofluorescein was prepared as reported by Haugland and coworkers with slight modifications.(2) Briefly, to a solution of 4-fluororesorcinol (328 mg, 2.98 mmol) in concentrated methanesulfonic acid (3 mL), tetrafluoroftalic anhydride (438 mg, 1.99 mmol) was added. The reaction mixture was heated under nitrogen at 80°C for 48 h. The mixture was then cooled down to RT and dropped into 7 volumes of ice water. The precipitate was recovered by filtration and dried under vacuum at 60°C for 2 hours. The ^1^H NMR showed the residual traces of methanesulfonic acid, the product was dissolved in EtOAc (50 mL) and washed 3 times with 50 mL of water. The organic layer was collected, dried over MgSO4, filtered, and the solvent removed under reduced pressure at 50°C, to give 2,4,5,7,7’-hexafluorofluorescein as an orange powder which was used for the following step without further purification (490 mg, 1.11 mmol, 56%).

^1^H NMR (400 MHz, DMSO-d_6_, 298 K) δ = 7.40 (s, 1H, Ar); 7.42 (s, 1H, Ar); 7.48 (s, 1H, Ar); 7.51 (s, 1H, Ar). ^19^F NMR (400 MHz, DMSO-d_6_, 298 K) δ = -140.98 (td, J=19.4, 9.3 Hz, 1F); -141.33 (dd, J=10.9, 7.7 Hz, 2F); -145.05 (t, J=17.5 Hz, 1F) , -145.68 (td, J=18.9, 9.1 Hz, 1F), -153.72 (ddd, J=20.1, 17.9, 4.4 Hz, 1F).

A solution of 2,4,5,6,7,7’-hexafluorofluorescein (490 mg, 1.11 mmol) in acetone (5 mL) was added to a solution of NaN_3_ (86.9 mg, 1.33 mmol) in water (1 mL) under stirring. The reaction mixture was heated under reflux for 20 hours. The progress of the reaction was monitored by TLC. At completion, acetone was removed under reduced pressure and the aqueous phase freeze-dried. The resulting residue was purified by flash chromatography (silicagel 60, 35-70 μm) using 100% EtOAc, then EtOAc/MeOH 1:1 (v/v) as the mobile phase. Relevant fractions were combined and the volatiles removed under vacuum to give the desired 6-azido-2,4,5,7,7’-pentafluorofluorescein (Oregon Green azide) as an orange solid (230 mg, 0.496 mmol, 45%).

^1^H NMR (400 MHz, MeOD, 298 K) δ = 6.79 (s, 1H, Ar); 6.81 (s, 1H, Ar); 6.95 (s, 1H, Ar); 6.98 (s, 1H, Ar). ^19^F NMR (400 MHz, MeOD, 298 K) δ = -131.81 (bs, 1F); -136.71 (bs, 2F); -145.29 (dd, J = 20.3, 14.9 Hz, 1F); -145.73 (dd, J = 20.6, 4.9 Hz, 1F).

FTIR (neat): ν 3416, 3228, 2361, 2140, 1617, 1557, 1497, 1339, 1300, 1150, 992, 843, 599 cm^-1^. Expected m/z for (M-H)^+^: 464.02. Found 464.00.

**Synthesis of fluorescently labelled glycopolymers (5)_Man_ and (5)_Gal_.**

To a solution of deprotected polymer (DP=73, 200 mg, 1.61 mmol of clickable alkyne units) in 10 mL of anhydrous DMF, oregon green azide (7.45 mg, 0.0161 mmol) and bipyridine (101 mg, 0.644 mmol) were added. The mixture was degassed by N_2_ bubbling for 15 minutes, Cu(I)Br (46.2 mg, 0.322 mmol) was quickly added under positive pressure of N_2_, and the resulting solution degassed again under nitrogen for further 15 minutes. The reaction mixture was stirred at room temperature for 72 hours and monitored by GPC with UV-vis (λ=496 nm) and RI detection. The solution was then split into 5 mL aliquots under nitrogen and each added of either 2’-azidoethyl-*O*-galactopyranoside or 2’- azidoethyl -*O*-mannopyranoside (401 mg, 1.61 mmol of each sugar azide). After degassing the solutions for 15 minutes under nitrogen, 100 µL of a 215 mg mL^-1^ sodium ascorbate (21.5 mg, 0.107 mmol) stock solution in water were added to each reaction mixture. The reaction was left under stirring at room temperature for 48 hours and monitored by ^1^H NMR following the appearance of the characteristic triazole ring signal as a broad singlet at around 8-8.2 ppm. Most of the DMF was then removed by precipitation in THF and the resulting solid residue was redissolved in water, transferred into dialysis bags (MWCO 3.5 kDa) and dialyzed in the dark against DI water. After 3 days the solutions were freeze-dried in the dark to obtain galactosylated **(5)_Gal_** or mannosylated (**5)_Man_** glycopolymers as pink-orange light solids.

**Figure C.** ^1^H NMR purified galactosylated Oregon Green-labelled glycopolymer **(5)_Gal_** in DMSO-d_6_.

**Figure D.** ^1^H NMR of purified mannosylated Oregon Green-labelled glycopolymer **(5)_Man_** in DMSO-d_6_.


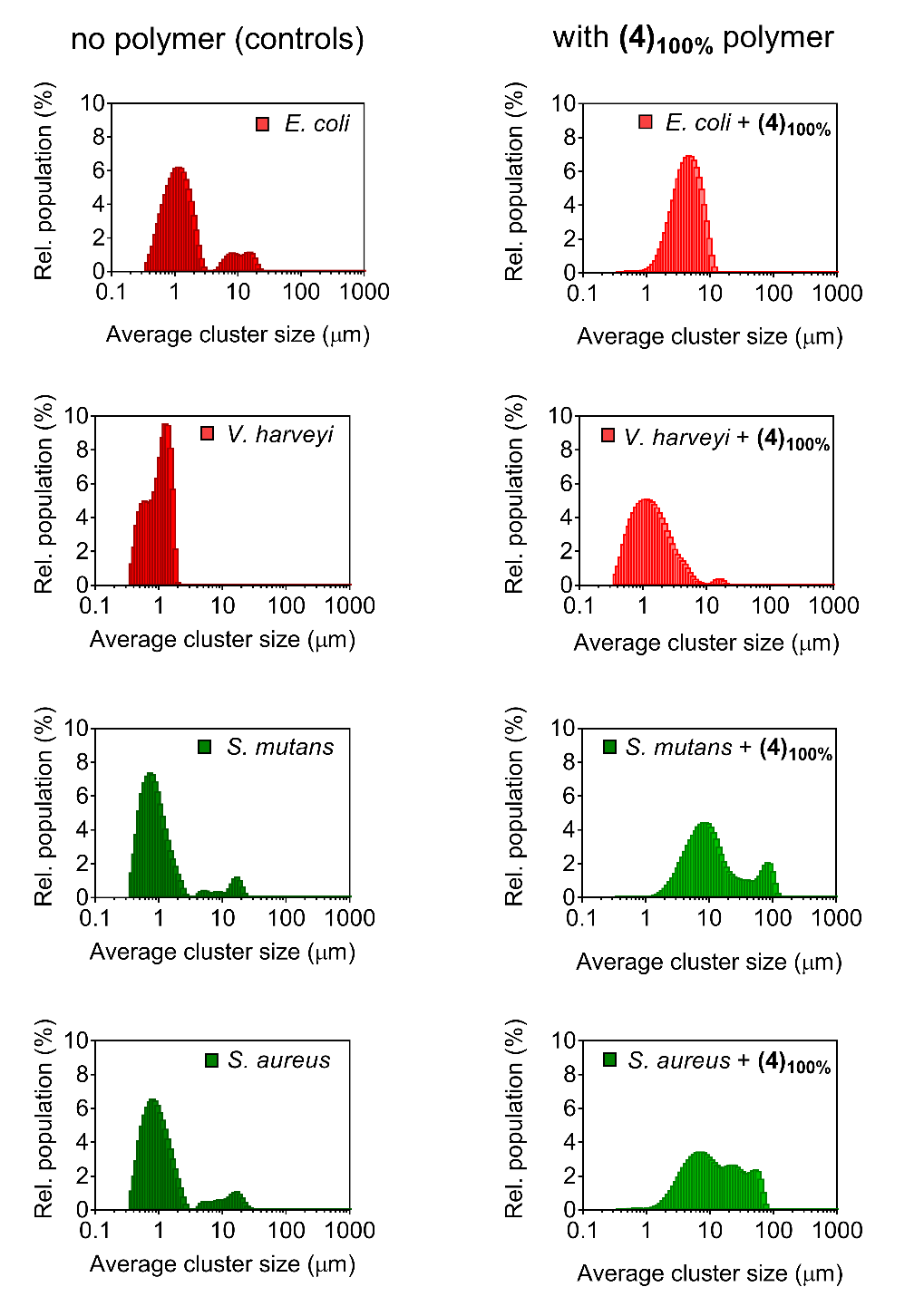


**Figure E.** Bacterial aggregation mediated as quantified *via* master sizer (Coulter counter) analysis of polymer–bacteria clusters: treatment with sulfobetaine polymer **(4)_100%_** (right column) *vs.* untreated bacteria controls (left column).

**References**

1. Ladmiral V, Mantovani G, Clarkson GJ, Cauet S, Irwin JL, Haddleton DM. Synthesis of Neoglycopolymers by a Combination of “Click Chemistry” and Living Radical Polymerization. Journal of the American Chemical Society. 2006;128(14):4823-30.

2. Sun W-C, Gee KR, Klaubert DH, Haugland RP. Synthesis of Fluorinated Fluoresceins. The Journal of Organic Chemistry. 1997;62(19):6469-75.
